# Cancer and Heart Disease Death Rates,*^,^^^†^^ Among Men and Women Aged 45–64 Years — United States, 1999–2018

**DOI:** 10.15585/mmwr.mm6921a4

**Published:** 2020-05-29

**Figure:**
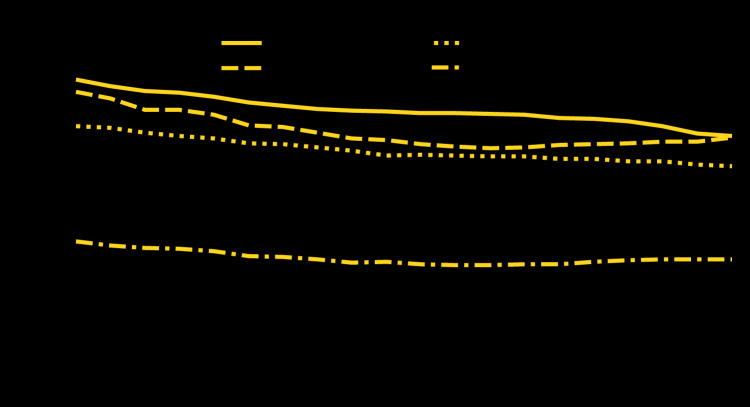
The cancer death rate for both men and women aged 45–64 years declined steadily from 247.0 per 100,000 in 1999 to 194.9 in 2018 for men and from 204.1 to 166.3 for women. The heart disease death rate for men declined from 1999 (235.7) to 2011 (183.5) but then increased to 192.9 in 2018. For women, the heart disease death rate declined from 1999 (96.8) to 2011 (74.9), increased through 2016 (80.3), and then leveled off. In 2018, the cancer death rate for men aged 45–64 years was 1% higher than the heart disease death rate; for women, the cancer death rate was approximately twice the heart disease death rate.

**Source: **National Vital Statistics System, Mortality Data. https://www.cdc.gov/nchs/nvss/deaths.htm.

